# Distal Femur Replacement: An Option for Osteoporotic Fractures in the Elderly

**DOI:** 10.7759/cureus.50762

**Published:** 2023-12-19

**Authors:** Kamran Hafeez, Gourav Garg, Mariette Anto, Vikram Desai

**Affiliations:** 1 Orthopaedics, Kings Mill Hospital, Sutton in Ashfield, GBR

**Keywords:** elderly trauma, osteoporotic fracture, distal femur replacement, periprosthetic femur fracture, distal femur fracture

## Abstract

Background

A distal femur fracture (DFF) around the native or prosthetic knee is commonly seen in the osteoporotic elderly population. Surgical management is required to restore the function. Fracture fixation requires a period of restricted weight-bearing; however, distal femoral replacement (DFR) allows immediate weight-bearing and quicker recovery.

Methods

All patients who underwent distal femur replacement from 2020 to 2023 at our hospital were retrospectively reviewed. Data related to the patient's demographics, medical comorbidities, preinjury mobility status, perioperative management and length of stay were collected.

Results

Eleven patients with 13 distal femoral replacements were included. There were 10 periprosthetic and 3 native fractures around the distal femur. Two patients had bilateral periprosthetic fractures. The median age was 84 years (range 62-95) with all patients being females. Eight patients were living in their homes while three were care home residents. The median duration of surgery was 120 min. The mean blood loss was 350 ml. Patients were mobilised out of bed at a median of three days and were able to walk for 2 meters with a frame at a mean of 10 days (range 3-15) except for two patients whose mobility was limited to the chair. The mean length of hospital stay was 32 days (range 8-54). All patients were discharged back to their original destination except for one who was shifted to a care home instead of her own home.

Conclusion

In our opinion, distal femur replacement provided a more favourable outcome with respect to pain management, early rehabilitation with full weight-bearing immediately following the surgery and fewer complications. Furthermore, in our hands, the surgical time was short with limited blood loss.

## Introduction

A distal femur fracture is a challenging problem in elderly patients. It significantly affects mobility. Osteoporosis is one of the important risk factors for these fractures [1]. Fragility fractures generally affect the proximal femur; however, they involve the distal femur in about 4-6% of the femoral fractures [2]. Most of these patients present with a history of low-energy trauma. It can be seen in native knees but increasingly becomes more common in patients already having total knee implants in place. There is a high-stress mismatch zone between osteoporotic bone and a metal component [3]. In addition, component malalignment and notching also increase the risk of periprosthetic fracture in this area [4].

The aim of management of these fractures is always to intervene as early as possible to mobilise and to lower complications related to prolonged immobilisation [5]. Treatment options include internal fixation or distal femoral replacement [6-8]. The challenge is poor bone stock and comminution, which makes it difficult to get good purchase of screws and bolts in the bone [9]. These limits internal fixation to allow early weight bearing. On the other hand, distal femoral replacement provides the apparent benefit of immediate stability, which allows early weight-bearing [10].

The aim of this study is to present our experience in the management of these complicated fractures.

This article's findings were previously presented as a poster at the British Indian Orthopaedic Society Conference held in London, United Kingdom, on 7th July 2023.

## Materials and methods

This was a retrospective study conducted in our hospital. We analysed retrospectively our electronic theatre record and identified the patients who were operated on for distal femur fractures either native or periprosthetic. All patients who underwent distal femoral replacement were included. We identified 11 patients who underwent distal femoral replacement from August 2020 to January 2023. Patients who were treated with internal fixation of fractures were excluded.

Medical case notes were retrieved from the medical record room and reviewed for all these patients. Records were assessed for the patient's demographics, mobility status prior to a fall, medical co-morbidities, mechanism of trauma, operative details and inpatient management. All patients were admitted through emergency. X-ray imaging was done, including knee and full-length femur views, to assess the extension and pattern of fracture. The CT scan was also done in a few patients in which fracture geometry on plain radiographs was not clear enough to decide. Most of the patients had multiple co-morbid medical conditions. Patients were evaluated by the orthogeriatric team and optimised medically before the operative procedure. All cases were discussed in multidisciplinary meetings with lower limb arthroplasty surgeons for possible options for management. There were three patients with a native distal femur fracture with osteoporosis and comminuted fractures. The fracture configuration of all these patients was deemed unsuitable for fixation and decided to be managed with distal femoral replacement. The rest of the patients had periprosthetic fractures around the femoral component. There was not enough bone stock around the femur component, which could be fixed with adequate stability.

Patients were operated by lower limb arthroplasty surgeons after medical optimisation and anaesthesia assessment. After anaesthesia, the patient was positioned supine on the operating table. A thigh tourniquet was applied and inflated during the initial part of the procedure. Anterior midline incision with a medial parapatellar approach was used to approach the fracture. A bony cut was made in the distal femur just above the fracture in the femur perpendicular to the anatomical axis. Fractured bone pieces and femoral condyles/implants were dissected all around and excised. The tibial surface was prepared in a routine knee arthroplasty fashion in native knees and the tibial implant was removed with minimal bone loss in patients with periprosthetic fractures. Femoral and tibial canals were reamed; the defect was measured and reconstructed with modular components using cemented intramedullary stems. Postoperatively patients were encouraged for range-of-motion exercises and mobilised with the help of a physiotherapist. Data were collected on a proforma, and descriptive statistical analysis was done with SPSS 16.0 (IBM Corp., Armonk, NY, USA).

## Results

There were 11 patients admitted with distal femur fractures treated with distal femoral replacement. Two patients had bilateral total knee replacement with periprosthetic fractures involving both of their knees. Among these fractures, there were 10 periprosthetic and 3 native fractures around the distal femur. Median age was 84 years (range 62-95). All patients being admitted were females. Eight patients were living in their homes, four with their families and four were living alone. Three patients were care home residents. All were independently mobile before admission, except one who was a care home resident and mobilised up to a chair with a few steps only. Eight patients were using a walker frame, two using a walking stick and one was independently mobile without any support (Table 1).

**Table 1:** Patient demographics HTN = Hypertension; DM = Diabetes mellitus; CKD = Chronic kidney disease; AF = Atrial fibrillation

S.No.	Age	Type of fracture	Residential home	Mobility status	Walking aid	Medical comorbid
1	73	Native	Bungalow	Outdoor	Frame	HTN, DM
2	89	Native	Bungalow	Within home	Frame	HTN
3	90	Periprosthetic	Bungalow	Within home	Frame	HTN, DM, CKD
4	80	Native	House	Within home	Frame	HTN
5	63	Periprosthetic	House	Outdoor	Stick	HTN, DM
6	83	Periprosthetic	Bungalow	Outdoor	Stick	HTN, Hypothyroid
7	90	Periprosthetic	House	Outdoor	Frame	HTN, DM, CKD
8	84	Periprosthetic	Care home	Bed to chair with few steps	Frame	Dementia
9	95	B/L Periprosthetic	Bungalow	Within home	Frame	HTN, DM, AF, CKD
10	62	Periprosthetic	Care home	Within home	None	DM
11	93	B/L Periprosthetic	Care home	Within home	Frame	DM

**Figure 1:**
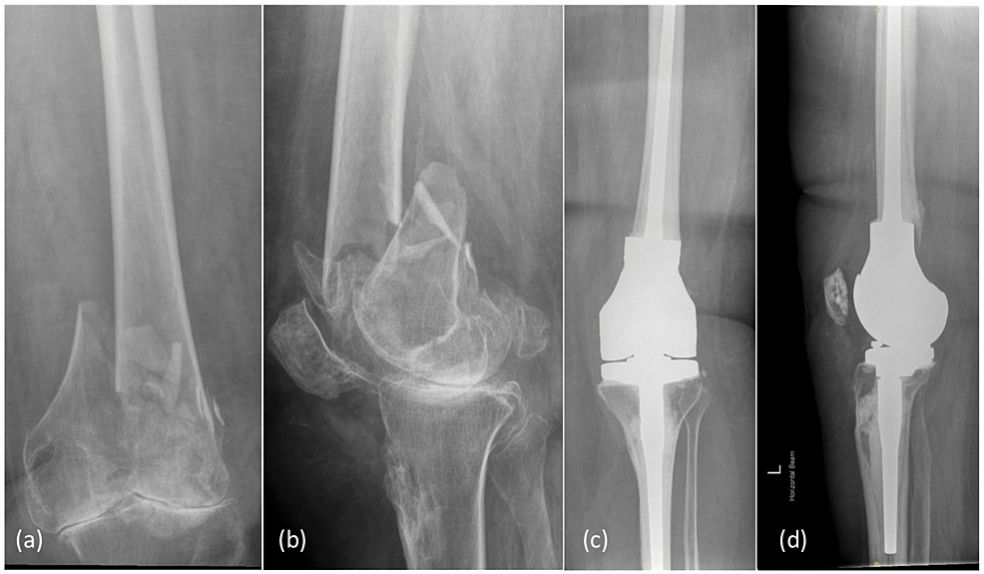
A 73-year-old lady with a distal femur comminuted fracture treated with distal femur replacement Preop xrays (a,b), Postop xrays (c,d)

Six procedures were done under spinal anaesthesia and seven were performed under general anaesthesia. The median duration of surgery was 120 min. The mean blood loss was 350 ml. One of the patients with bilateral periprosthetic fractures was operated on in a single setting for both knees while the other was operated on as a staged procedure. A patient who was operated on in a single stage developed bone implantation syndrome. She survived but her post-op rehabilitation was delayed. Patients were reviewed by physiotherapists in the postop period. Patients were allowed for full weight-bearing. Patients were mobilised out of bed at a median of three days and were able to walk for 2 meters with a frame at a mean of 10 days (range 3-15) except for two patients whose mobility was limited to the chair. The mean length of stay was 32 days (range 8-54). All patients were discharged back to their original destination except for one who was shifted to a care home instead of her own home. There was no inpatient mortality. Pre and postop X-rays of one of the patients are shown in Figure 1.

## Discussion

In our study, distal femur replacement in complex osteoporotic fractures allowed immediate weight-bearing in the postop period. Most of the patients were discharged to their residences from the hospital.

A distal femur fracture in the elderly affects mobility and is associated with significant medical complications. Traditionally, these fractures have been managed with fixation. Patients are required to keep non-weight bearing till fractures show some signs of healing. These fractures take a longer time to heal and are associated with a high rate of complications. Moloney GB et al. retrospectively analysed distal femur fractures managed in their hospitals with lateral-based locking plates. They reported a 24% non-union rate in their series and 38% experienced one systemic complication [11].

Fracture healing is always a challenge in the elderly age group due to osteoporosis and the increased frequency of associated co-morbidities. In displaced neck of femur fractures, arthroplasty remains a preferable option as compared to fixation [12,13]. In recent years, some studies suggest distal femur replacement is a promising option in the management of osteoporotic distal femur fractures and allows early mobilisation [14,15]. It prevents complications related to prolonged immobilisation and revision surgery in case of failure of union [16].

Saidi et al., in their series, compared distal femur replacement as an option for periprosthetic fractures with allograft composite reconstruction and revision with augments [17]. They reported decreased blood loss and shorter operating time and hospital stays in patients having distal femur replacement. Tibbo et al. compared the outcomes between distal femoral replacement and open reduction and internal fixation of comminuted distal femur fractures [18]. They found a higher blood loss (592 ml vs 364 ml) and length of stay (13 days vs 6.5 days) among the distal femur replacement group. In our study, the mean blood loss was 350 ml for patients undergoing distal femur replacement. However, unfortunately, we didn’t have any comparison group. Our mean length of stay was higher (32 days), as most of them were waiting for their care packages but were discharged back to their initial residence except for one who was shifted to a care home instead of his home.

Wadhwa et al., in their meta-analysis including 58 studies, compared distal femur replacement and open reduction internal fixation for functional outcome and complications and found no difference [19]. Lex JR et al., in their systematic review, reported no difference in terms of mortality and reoperation rate among distal femoral replacement and open reduction internal fixation for periprosthetic fractures [20]. However, they suggested distal femur replacement to be more reliable in cases of comminuted fractures deemed unsuitable for fixation. The authors suggest future prospective randomised trials to further assess the indications for distal femur replacement in the management of these complex fractures.

There were some limitations in our study, including the small number of patients, single-institution study, and no comparison. group.

## Conclusions

In our opinion, a distal femur replacement in these complicated osteoporotic fractures around the knee in the elderly provided a more favourable outcome with respect to pain management, early rehabilitation with full weight-bearing immediately following the surgery and fewer complications. Furthermore, in our hands, the surgical time was short with limited blood loss. We recommend large-scale studies in the future with an increased number of patients receiving distal femoral replacement and the inclusion of a comparison group of patients undergoing fracture fixation for a better understanding of the efficiency of this surgical intervention.

## References

[1] Ricci WM (2015). Periprosthetic femur fractures. J Orthop Trauma.

[2] Canton G, Giraldi G, Dussi M, Ratti C, Murena L (2019). Osteoporotic distal femur fractures in the elderly: peculiarities and treatment strategies. Acta Biomed.

[3] Singh JA, Jensen M, Lewallen D (2013). Predictors of periprosthetic fracture after total knee replacement: an analysis of 21,723 cases. Acta Orthop.

[4] Yoo JD, Kim NK (2015). Periprosthetic fractures following total knee arthroplasty. Knee Surg Relat Res.

[5] Ehlinger M, Ducrot G, Adam P, Bonnomet F (2013). Distal femur fractures. Surgical techniques and a review of the literature. Orthop Traumatol Surg Res.

[6] Herrera DA, Kregor PJ, Cole PA, Levy BA, Jönsson A, Zlowodzki M (2008). Treatment of acute distal femur fractures above a total knee arthroplasty: systematic review of 415 cases (1981-2006). Acta Orthop.

[7] Ebraheim NA, Kelley LH, Liu X, Thomas IS, Steiner RB, Liu J (2015). Periprosthetic distal femur fracture after total knee arthroplasty: a systematic review. Orthop Surg.

[8] Konan S, Sandiford N, Unno F, Masri BS, Garbuz DS, Duncan CP (2016). Periprosthetic fractures associated with total knee arthroplasty: an update. Bone Joint J.

[9] Berend KR, Lombardi AV Jr (2009). Distal femoral replacement in nontumor cases with severe bone loss and instability. Clin Orthop Relat Res.

[10] Rahman WA, Vial TA, Backstein DJ (2016). Distal femoral arthroplasty for management of periprosthetic supracondylar fractures of the femur. J Arthroplasty.

[11] Moloney GB, Pan T, Van Eck CF, Patel D, Tarkin I (2016). Geriatric distal femur fracture: are we underestimating the rate of local and systemic complications?. Injury.

[12] Ravikumar KJ, Marsh G (2000). Internal fixation versus hemiarthroplasty versus total hip arthroplasty for displaced subcapital fractures of femur--13 year results of a prospective randomised study. Injury.

[13] Rogmark C, Carlsson A, Johnell O, Sernbo I (2002). A prospective randomised trial of internal fixation versus arthroplasty for displaced fractures of the neck of the femur. Functional outcome for 450 patients at two years. J Bone Joint Surg Br.

[14] Hart GP, Kneisl JS, Springer BD, Patt JC, Karunakar MA (2017). Open reduction vs distal femoral replacement arthroplasty for comminuted distal femur fractures in the patients 70 years and older. J Arthroplasty.

[15] Bettin CC, Weinlein JC, Toy PC, Heck RK (2016). Distal femoral replacement for acute distal femoral fractures in elderly patients. J Orthop Trauma.

[16] Nauth A, Ristevski B, Bégué T, Schemitsch EH (2011). Periprosthetic distal femur fractures: current concepts. J Orthop Trauma.

[17] Saidi K, Ben-Lulu O, Tsuji M, Safir O, Gross AE, Backstein D (2014). Supracondylar periprosthetic fractures of the knee in the elderly patients: a comparison of treatment using allograft-implant composites, standard revision components, distal femoral replacement prosthesis. J Arthroplasty.

[18] Tibbo ME, Parry JA, Hevesi M, Abdel MP, Yuan BJ (2022). Distal femoral replacement versus ORIF for severely comminuted distal femur fractures. Eur J Orthop Surg Traumatol.

[19] Wadhwa H, Salazar BP, Goodnough LH (2022). Distal femur replacement versus open reduction and internal fixation for treatment of periprosthetic distal femur fractures: a systematic review and meta-analysis. J Orthop Trauma.

[20] Lex JR, Di Michele J, Sepehri A, Chuang TC, Backstein DJ, Kreder HJ (2022). Distal femoral replacement or internal fixation for management of periprosthetic distal femur fractures: A systematic review. Knee.

